# BioContainers: an open-source and community-driven framework for software standardization

**DOI:** 10.1093/bioinformatics/btx192

**Published:** 2017-03-30

**Authors:** Felipe da Veiga Leprevost, Björn A Grüning, Saulo Alves Aflitos, Hannes L Röst, Julian Uszkoreit, Harald Barsnes, Marc Vaudel, Pablo Moreno, Laurent Gatto, Jonas Weber, Mingze Bai, Rafael C Jimenez, Timo Sachsenberg, Julianus Pfeuffer, Roberto Vera Alvarez, Johannes Griss, Alexey I Nesvizhskii, Yasset Perez-Riverol

**Affiliations:** 1 Department of Pathology, University of Michigan, Ann Arbor, MI, USA; 2 Bioinformatics Group, Department of Computer Science, Albert-Ludwigs-University Freiburg, Freiburg, Germany; 3 Albert-Ludwigs-University, Department of Computer Science, Bioinformatics Group, Freiburg, Baden-Württemberg, Freiburg, Freiburg; 4 Wageningen Plant Research, Cluster Bioinformatics, Wageningen, AD, Gelderland, Netherlands; 5 Department of Genetics, Stanford University, USA; 6 Medizinisches Proteom-Center, Ruhr-University Bochum, Germany; 7 Proteomics Unit (PROBE), Department of Biomedicine, University of Bergen, Bergen, Norway; 8 Computational Biology Unit (CBU), Department of Informatics, University of Bergen, Bergen, Norway; 9 KG Jebsen Center for Diabetes Research, Department of Clinical Science, University of Bergen, Norway; 10 KG Jebsen Center for Diabetes Research, Department of Clinical Science, University of Bergen, Bergen, Norway; 10a Center for Medical Genetics and Molecular Medicine, Haukeland University Hospital, Bergen, Norway; 11 EMBL Outstation, European Bioinformatics Institute, Proteomics Services, Wellcome Trust Genome Campus, Hinxton, Cambridge, UK; 12 Computational Proteomics Unit and Cambridge Centre for Proteomics, Department of Biochemistry, University of Cambridge, Cambridge, UK; 13 Universität Tübingen, Wilhelm Schickard Institut für Informatik, Applied Bioinformatics Group, Tübingen, Germany; 14 Eberhard-Karls-Universität Tübingen, Department of Computer Science, Applied bioinformatics, Tübingen; 15 Computational Biology Branch, National Center for Biotechnology Information, National Library of Medicine, National Institutes of Health, Bethesda, MD, USA; 16 Division of Immunology, Allergy and Infectious Diseases, Department of Dermatology, Medical University of Vienna, Austria; 17 Department of Computational Medicine and Bioinformatics, University of Michigan, Ann Arbor, MI, USA

## Abstract

**Motivation:**

BioContainers (biocontainers.pro) is an open-source and community-driven framework which provides platform independent executable environments for bioinformatics software. BioContainers allows labs of all sizes to easily install bioinformatics software, maintain multiple versions of the same software and combine tools into powerful analysis pipelines. BioContainers is based on popular open-source projects *Docker* and *rkt* frameworks, that allow software to be installed and executed under an isolated and controlled environment. Also, it provides infrastructure and basic guidelines to create, manage and distribute bioinformatics containers with a special focus on omics technologies. These containers can be integrated into more comprehensive bioinformatics pipelines and different architectures (local desktop, cloud environments or HPC clusters).

**Availability and Implementation:**

The software is freely available at github.com/BioContainers/.

## 1 Introduction

Bioinformatics have emerged as a crucial contributor to our understanding of the function and behavior of systems biology with the development of novel algorithms, the connection of various tools into complex pipelines (Perez-Riverol *et al.*, 2014) and their deposition and dissemination. These developments have been moved from single and individual tools to complex and integrated workflow systems such as OpenMS (Röst *et al.*, 2016), Taverna (Wolstencroft *et al.*, 2013) and Galaxy (Afgan *et al.*, 2016), creating two major challenges for software developers and the bioinformatics community: (i) software availability and (ii) reproducible experiments. Several algorithms software and pipelines in bioinformatics require substantial effort for correct installation and configuration (e.g. conflicting system dependencies). A good starting point for the replicability and reproducibility of the original results should be well-documented (software parameters, dependencies, etc.) and easily installable software (Leprevost *et al.*, 2014). Container based technologies such as Docker (docker.com) or rkt (https://coreos.com/rkt) have emerged to overcome these challenges by automating the deployment of applications inside so-called software *containers*. A software container provides an isolated environment for the installation and execution of a specific software, without affecting other parts of the system. Different groups have proposed the use of Docker containers to solve bioinformatics problems (Belmann *et al.*, 2015; Moreews *et al.*, 2015). However, most of these projects have been limited to individual efforts and only explore the potential of Docker technology in bioinformatics.

In this manuscript, we present BioContainers (biocontainers.pro), a community-driven project that provides the infrastructure and guidelines to create, manage and distribute bioinformatics containers. The BioContainers architecture facilitates the requests and maintenance of bioinformatics containers, and the interaction between the users and the community. With more than 30 contributors, the community-driven approach guarantees the sustainability and scalability of the project. In addition, BioContainers has been integrated with the BioConda (https://bioconda.github.io/) project enabling the automatic generation of containers for each BioConda recipe. At the time of writing, BioContainers provides more than 2076 containers that can be searched, tagged and accessed through a common web registry (biocontainers.pro/registry/). Finally, we discuss the integration of BioContainers as a container provider with other open-source projects such Galaxy (https://galaxyproject.org/) and PhenoMeNal H2020 (http://phenomenal-h2020.eu/home/).

## 2 BioContainers architecture

**Fig. 1:**
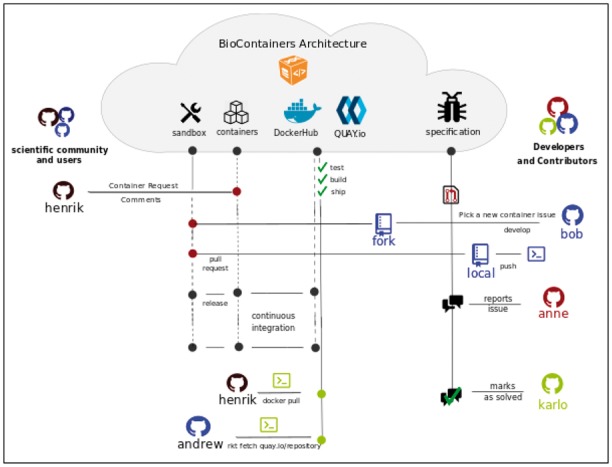
Overview of the BioContainers architecture: Users and developers can use the BioContainers infrastructure by interacting via GitHub account page. All container Dockerfiles are freely available and people are encouraged to participate submitting pull requests or asking for new containerized software. Containers can be acquired via Docker command line interface, or by downloading the Dockerfile directly from the GitHub organization

The BioContainers architecture is built on two main components: (i) a GitHub organization (github.com/BioContainers/) including all Dockerfiles (for the Dockerfile-based containers), the specification, and tools to create/manage containers; (ii) the BioContainers registries and Registry-UI (biocontainers.pro/registry/) where the available containers are built by an automatic system and made available for download, ready-to-use, by the Docker or rkt (see example, http://biocontainers.pro/docs/101/running-example/). Figure 1 shows the BioContainers infrastructure from the user request to the final deployment of the container.

Users of BioContainers can request a software container by opening an issue in the container’s repository containing information about software (name, URL or binary to be packaged). A member of the BioContainers community will pick up the issue and generate the specific container. An automated build system is configured/deployed making the new container available within hours. To integrate both registries we developed a Registry-UI (biocontainers.pro/registry/) that allows users to search, tag and find BioContainers independently of where they have been deployed. The user can then use docker or rkt to pull or fetch the corresponding container:

$>dockerpullbiocontainers/blast

$>dockerrun-v/home/user/workplace:/data/

biocontainers/blastblastp-queryseq.fa-db

zebrafish.fa

## 3 Dockerfile-based and mulled containers

In order to create and build a new container, the BioContainer developers can follow two approaches: (i) create a BioConda recipe for the software or (ii) create a Dockerfile recipe in the container’s repository (http://github.com/BioContainers/containers). In the first approach the developer should create a BioConda recipe following the BioConda guidelines (https://bioconda.github.io/guidelines.html). A container generation tool (https://github.com/BioContainers/auto-mulled/) automatically creates a container for the BioConda package and pushes it into BioContainers quay.io registry. These ‘mulled containers’ are generated using the *involucro* tool (https://github.com/involucro/involucro) which enables the generation of containers without any Dockerfile definition, reusing already existing recipes from other package managers, like Conda or Alpine. In summary, involucro will install the given (Conda) package into a build-time container which has the the preferred package manager already installed and copies the resulting new image layer on top of a runtime environment defined by BioContainers (busybox). (ii) In the second approach, a recipe file must be named Dockerfile which holds all the instructions necessary for creating the complete container. As part of the project specifications, we are providing a template for developers to ‘containerize’ their own applications (https://github.com/BioContainers/specs/blob/master/container-specs.md). For each BioContainers the developer should provide metadata about the software such as the name, version, license, web-page and the maintainer. Both strategies are already aligned and the metadata needed to create a BioConda recipe in the YAML file is the same we recommended for the Dockerfile. This metadata enables BioContainers to find, describe and maintain each containers following best practices (Leprevost *et al.*, 2014).

## 4 Tools and future directions

At the time of writing, BioContainers provides more than 2076 containers ready to be used. The integration with the BioConda project (bioconda.github.io) has enabled us to create a new type of containers without any Dockerfile, reusing already existing BioConda recipes. The Galaxy Project has recently proposed Docker containers as a new way to solve workflow dependencies (biocontainers.pro/docs). Also, the PhenoMeNal H2020 project has adopted and implemented BioContainers guidelines and deploying their containers into the BioContainers architecture. The BioContainers community is now working on new ways for testing containers and for workflow/pipelines integration.

## Funding

F.V.L. and A.I.N are supported by NIH grant numbers R01-GM-094231 and U24-CA-210967 (to A.I.N). H.L.R. is supported by the Swiss National Science Foundation (SNSF grant P2EZP3 162268) and EMBO (ALTF 854-2015). H.B. is supported by the Bergen Research Foundation and the Research Council of Norway. T.S., J.P. and J.U. acknowledge funding from BMBF (de.NBI, grant nos. FKZ 031 A 535A and FKZ 031 A 534A). Y.P-R. is supported by US NIH BD2K grant [U54 GM114833]. LG is supported by the BBSRC Strategic Longer and Larger grant (Award BB/L002817/1). P.M. is supported by EC Horizon 2020 grant agreement 654241.

*Conflict of Interest*: none declared.
